# New regression equations for predicting human teeth sizes

**DOI:** 10.1186/s13005-015-0067-8

**Published:** 2015-03-25

**Authors:** Vanessa Paredes, Beatriz Tarazona, Natalia Zamora, Rosa Cibrian, Jose Luis Gandia

**Affiliations:** Orthodontics Department, Faculty of Dentistry and Medicine, University of Valencia, Gasco Oliag nº1, 46010 Valencia, Spain; Physiology Department, Faculty of Medicine and Dentistry, University of Valencia, Spain, Valencia, Spain

**Keywords:** Moyers, Prediction, Regression equations, Tanaka-Johnston

## Abstract

**Introduction:**

The aims of the study were; to evaluate the applicability of the Moyers and Tanaka-Johnston Methods to individuals with a Spanish ancestry, to propose new regression equations using the lower four permanents incisors as predictors for the sum of the widths of the lower permanent canine and premolars, and to compare the new data to those from other populations.

**Methods:**

A total of 359 Spanish ancestry adolescents were selected. Their dental casts were measured using a 2D computerized system. Real teeth measurements were compared with those predicted using Moyers probability tables and Tanaka and Johnston equations, and standard regression equations were then developed.

**Results:**

Results showed that Upper and Lower Canine and Premolar (UCPM, LCPM) predictions are quite different depending on the used method. Moyers tables can only be validly applied to a 75% percentile for the mandible in both, males and females, 85% in males and 90-92% in females.

**Conclusions:**

Moyers predictions tend to underestimate UCPM and LCPM whereas Tanaka-Johnston predictions tend to overestimate them. Equations for estimating the combined width of the unerupted canine and premolars were; Male: UCPM = 12.68 + 0.42 LI and LCPM = 11.71 + 0.44 LI. Female: UCPM = 12.06 + 0.43LI and LCPM = 10.71 + 0.46 LI.

## Introduction

Predicting unerupted tooth size of Upper and Lower Canine and Premolars (UCPM, LCPM) in mixed dentition is important for a good diagnosis and for choosing a therapy [1]. To date, three basic groups have been used to determine the mesiodistal widths of unerupted canines and premolars.

1- Analyses based on correlation and regression equations, expressed as prediction tables. Both Moyers’ regression scheme [2] and Tanaka and Johnston’s equations [3] have achieved widespread clinical acceptance because of their simplicity and ease of application. 2- Analyses based on measurements taken from radiographs [4,5] of unerupted teeth. 3- Analyses based on a combination of correlations and regression equations and measurements on radiographs [6-8].

However, bearing in mind that these prediction methods are based on individuals of North American ancestry, it is not appropriate to use them on different populations of different biological origin. For this reason, several linear regression equations have been proposed for different populations [9-23].

Odontometric data from Spanish ancestry children are not so widely available and, to date, there is no study in the literature examining the accuracy of Moyers probability tables and Tanaka and Johnston equations in predicting the size of unerupted canines and premolars in a Spanish ancestry sample. The aims of the present study were, therefore, to evaluate the applicability of the Moyers and Tanaka-Johnston methods to Spanish ancestry individuals; to propose new regression equations using the lower four permanent incisors as predictors of the sum of the widths of the lower and upper permanent canine and premolars; and to compare the new data with those of other populations.

## Material and methods

500 patients attending the Orthodontics Department of the University of Valencia, Spain were chosen. Subjects presented to the orthodontic clinic in sequential order over a fixed period of time (January 2010-January 2012). A retrospective study was carried out and approved by the Ethics Committee of Research into Humans of the Experimental Research Ethics Committee at Valencia University, Spain. Reference number H1373014083626. All patients whose records were used in this work received detailed information about the study, reflected on an informed consent. There was also a confidentiality agreement stating that patients’ personal data and their records would only be used for scientific purposes.

In order to predict unerupted teeth sizes under the best conditions, patient selection criteria were:

- Permanent dentition from first molar to first molar.
- Lower and upper first molar totally erupted and without the gingiva overlapping the distal surface of the tooth.
- Good quality casts.
- No tooth agenesis or extractions.
- No previous orthodontic treatment.
- No restorations or teeth with anomalous shapes that could change the mesiodistal diameter of the tooth or bruxism.
- Spanish ancestors from at least 1 previous generation (Spanish means people living in Spain, Europe with at least 1 previous generation of Spanish ancestors).
- Class I relationship with no arch discrepancy.

The Spanish ancestry sample finally included 359 patients (169 = 47.1% males and 190 = 52.9% females), with a mean age of 14.8 years (range 11.2-19.2) similar for both sex.

The power analysis showed that 359 patients were needed to achieve 90% power to detect clinically meaningful differences of the values. To compensate for possible dropouts during the study, more patients were we enrolled.

All the study casts were digitized with a conventional scanner and calibrated before any measurement was taken, using a simple method. In this calibration system, dental casts were surrounded by millimeter paper sheet. When the arches have been digitized, the magnification of the millimeter paper in the two axes is known and the dental cast magnification can be calculated [24]. A 2D digital software program designed by the University of Valencia, previously tested and found to be accurate and reliable [16], was used to determine dental sizes (in millimeters) of the lower four permanent incisors. With the aid of the mouse as a user interface, mesiodistal size of each permanent tooth on the image of the casts was marked. The software determines dental sizes in millimeters automatically.

The Tanaka and Johnston [3] equations used are as follow;

$$ \begin{array}{l}1/2\mathrm{M}\mathrm{D}\ \mathrm{Lower}\ \mathrm{I}\mathrm{ncisors}\left(\mathrm{L}\mathrm{I}\right)\mathrm{width}+10.5\mathrm{mm}=\mathrm{Estimated}\ \mathrm{LCPM}\ \mathrm{width}\\ {}1/2\mathrm{M}\mathrm{D}\ \mathrm{Lower}\ \mathrm{I}\mathrm{ncisors}\left(\mathrm{L}\mathrm{I}\right)\mathrm{width}+11.0\mathrm{mm}=\mathrm{Estimated}\ \mathrm{UCPM}\ \mathrm{width}\end{array} $$

### Statistical analysis

All statistical analyses were performed using the SPSS© Vs. 10.0 Inc 1989–1999 Copyright, statistics package for Windows.

The descriptive analysis provides the relevant statistics for primary analysis variables: the mesiodistal sizes of the lower incisors (LI), the upper canine-premolars (UCPM) and lower canine-premolars (LCPM). The two latter ones are calculated as a mean of those recorded on both sides of each arch.

To evaluate the predictive power of the Moyers table, differences were calculated between the real values of those parameters (UCPM, LCPM) in the sample and those predicted by tables for percentiles in accordance with LI values. Likewise, the differences between the real values of the UCPM, LCPM and the values predicted by the Tanaka-Johnston [3] formula were calculated. For all of them, basic descriptive statistics and confidence levels of 95% are provided. All the mentioned information is segmented by sex, as sexual dimorphism is a key aspect of this investigation.

Regarding the inferential analysis undertaken, unpaired Student t-tests were applied to compare the mean equality hypothesis of UCPM and LCPM in males and females. The Student t-test for paired samples was applied to reach a conclusion over the equality of real mean values and estimated values, whether those of the Moyers’ tables [2] or the Tanaka-Johnston equation [3]. Assumptions regarding normality of parameters and homogeneity of variances were checked by means of Kolmogorov-Smirnov and Levene´s test respectively.

A simple linear regression analysis was developed to estimate, through least squares, the equation that relates the UCPM and LCPM to LI, in men and women. Correlation coefficients (r) and regression equations (y = a + bx) were formulated to evaluate the relationship between the summed widths of the 4 LI in millimeters (x, independent variable) and the canines and premolars (y, dependent variable), “a” the slope and “b” the intercept of each dental arch. Constants “a” and “b” in the standard linear regression equations (y = a + bx), determination coefficients (r^2^), and the standard errors of the estimates (SEE) were calculated for combined sexes and for each sex separately. The r^2^ value indicates the predictive accuracy of the regression equation for y based on values of x. Hypothesis of normality; homoscedasticity and no autocorrelation of residuals were checked. Re-estimation of equations was carried up in 75% of the sample in order to check its acceptance at an independent sample (25% remaining).

## Results

The reproducibility of the digital method was analysed by determining intra- and inter-examiner measurement errors, calculated by coefficients of variation (CVs = standard deviation- 100/mean) expressed as a percentage. Twenty dental casts from the present study were randomly selected. The measurements of the twenty dental casts were again determined by the same examiner (VP) (intra-examiner error) and by two different examiners (RC and JLG) (inter-examiner error) in order to obtain the CV. All CVs were very low (below 5.8 per cent) and similar between examiners. Digital methods CVs were 0.05 – 2.88 and 0.16 – 5.70 per cent for intra- and inter-examiner calibrations, respectively.

Since right and left side values are highly correlated (and non-independent) within individuals, mean of right and left side values was chosen in these statistical comparisons. Table 1 presents descriptive information on LI, UCPM and LCPM, sizes segmented by sex.

**Table 1:** **Mesiodistal Lower Incisor (LI), Upper Canine and Premolar (UCPM) and Lower Canine and Premolar (LCPM) tooth sizes per sex**

			**N**	**Mean ± SD(mm)**	**Minimum**	**Maximum**	**S. Error**	**CI 95%**	**P value**
**LI**	**Sex**	**T**	359	23.04 ± 1.45	17.77	26.39	.08	22.89 – 23.19	
		**M**	169	23.04 ± 1.46	17.77	26.39	.11	22.82 – 23.26	n.s.
		**F**	190	23.03 ± 1.44	19.39	26.11	.10	22.83 – 23.24	
**UCPM**		**T**	359	22.11 ± 1.07	19.49	25.49	.06	21.99 – 22.22	
		**M**	169	22.31 ± 1.06	19.68	25.25	.08	22.15 – 22.47	**
		**F**	190	21.92 ± 1.04	19.49	25.49	.08	21.77 – 22.07	
**LCPM**		**T**	359	21.60 ± 1.12	18.37	24.62	.06	21.48 – 21.71	
		**M**	169	21.82 ± 1.11	19.35	24.62	.09	21.65 – 21.99	**
		**F**	190	21.40 ± 1.09	18.37	24.47	.08	21.25 – 21.56	

t-Test of independent samples for assessing homogeneity of measurements per sex. n.s = notsignificant; ** = p < 0.01. Male + Female(Total); Male(M) and Female(F).

The first method used for prediction was Moyers’ tables. Table 2 provides the descriptive statistics for the difference in mm between the real values and those estimated by Moyers for the UCPM and LCPM, for the different percentile levels. For this analysis, those individuals whose LI values were either below or above the Moyers’ limits (1 and 10 respectively) were excluded, a margin of 0.25 mm. being accepted. Hence the effective sample in this section consisted of 348 cases. Moyers’ values systematically tend to underestimate the real values in the Spanish population.

**Table 2:** **The difference(mm) between the mean values of real Upper and Lower Canine and Premolar (UCPM, LCPM) tooth sizes and those predicted from Moyers’ charts per sex**

**Percentile level %**		**Sex**								
		**T**			**M**			**F**		
		**N**	**Mean difference ± SD(mm)**	**P value**	**N**	**Mean difference ± SD**	**P**	**N**	**Mean difference ± SD**	**P**
UCPM	**5**	348	2.65 ± 0.95	***	161	2.24 ± 0.87	***	187	3.00 ± 0.87	***
	**15**		2.06 ± 0.92	***		1.73 ± 0.87	***		2.35 ± 0.87	***
	**25**		1.73 ± 0.91	***		1.43 ± 0.87	***		1.98 ± 0.87	***
	**35**		1.44 ± 0.90	***		1.16 ± 0.87	***		1.68 ± 0.87	***
	**50**		1.08 ± 0.90	***		0.84 ± 0.87	***		1.28 ± 0.87	***
	**65**		0.72 ± 0.89	***		0.53 ± 0.87	***		0.88 ± 0.87	***
	**75**		0.43 ± 0.88	***		0.27 ± 0.87	***		0.57 ± 0.86	***
	**85**		0.10 ± 0.87	*		−0.03 ± 0.87	n.s.		0.21 ± 0.86	**
	**95**		−0.49 ± 0,.87	***		−0.55 ± 0.87	***		−0.44 ± 0.86	***
LCPM	**5**	348	2.74 ± 0.88	***	161	2.65 ± 0.90	***	187	2.81 ± 0.86	***
	**15**		2.02 ± 0.88	***		1.95 ± 0.90	***		2.07 ± 0.86	***
	**25**		1.58 ± 0.88	***		1.51 ± 0.90	***		1.64 ± 0.86	***
	**35**		1.24 ± 0.88	***		1.16 ± 0.90	***		1.30 ± 0.86	***
	**50**		0.79 ± 0.88	***		0.72 ± 0.90	***		0.84 ± 0.86	***
	**65**		0.32 ± 0.88	***		0.26 ± 0.90	***		0.37 ± 0.86	***
	**75**		−0.01 ± 0.88	n.s.		−0.06 ± 0.90	n.s.		0.03 ± 0.86	n.s.
	**85**		−0.44 ± 0.88	***		−0.50 ± 0.90	***		−0.39 ± 0.86	***
	**95**		−1.18 ± 0.88	***		−1.24 ± 0.90	***		−1.12 ± 0.86	***

t-Test of dependent samples for assessing homogeneity of measurements between the real values of the sample and those predicted by Moyers. n.s = not significant; * = p < 0.05; ** = p < 0.01; *** = p < 0.001. Male + Female(Total); Male(M) and Female(F).

The second method used was Tanaka-Johnston regression equations. For that, the difference between the real value and the predicted value was calculated using these equations for the UCPM and LCPM sizes (Table 3). In contrast to Moyers’ tables, these equations tend to overestimate the real values of the UCPM and LCPM sizes in the Spanish population. Thirdly, estimation from an own regression equation was proposed. Table 4 summarises the results of the 6 regression models undertaken: total maxilla, male maxilla, female maxilla, total mandible, male mandible and female mandible. The equations for estimating the combined width of the unerupted canine and premolars were:

$$ \begin{array}{l}\mathrm{Males}:\mathrm{UCPM}=12.68+0.42\mathrm{L}\mathrm{I}\ \mathrm{and}\ \mathrm{LCPM}=11.71+0.44\mathrm{L}\mathrm{I}\\ {}\mathrm{Females}:\mathrm{UCPM}=12.06+0.43\mathrm{L}\mathrm{I}\ \mathrm{and}\ \mathrm{LCPM}=10.71+0.46\mathrm{L}\mathrm{I}\end{array} $$

**Table 3:** **The difference (mm) between the mean values of real Upper and Lower Canine and Premolar(UCPM, LCPM) tooth sizes and those predicted from Tanaka-Johnston’s equations per sex**

	**Sex**								
	**T**			**M**			**F**		
	**N**	**Mean difference ± SD(mm)**	**P**	**N**	**Mean ± SD**	**P**	**N**	**Mean ± SD**	**P**
**UCPM**	359	−0.41 ± 0.88	***	169	−0.21 ± 0.88	**	190	−0.59 ± 0.85	***
**LCPM**		−0.42 ± 0.91	***		−0.20 ± 0.91	**		−0.61 ± 0.86	***

t-Test of dependent samples for assessing homogeneity of measurements between the realand predicted values of the sample. n.s = not significant; ** = p < 0.01; *** = p < 0.001.Male + Female(Total); Male(M) and Female(F).

**Table 4:** **Regression parameters for predictions of UCPM and LCPM tooth sizes in each arch and per sex**

			**Constants**				
			**r**	**a**	**b**	**SEE**	**r** ^**2**^
**UCPM**	**T**		0.574	12.34***	0.42***	0.87	0.330
	**Sex**	**M**	0.574	12.68***	0.42***	0.87	0.330
		**F**	0.592	12.06***	0.43***	0.84	0.351
**LCPM**	**T**		0.587	11.17***	0.45***	0.91	0.345
	**Sex**	**M**	0.577	11.71***	0.44***	0.91	0.333
		**F**	0.616	10.71***	0.46***	0.86	0.379

r (Pearson linear regression coefficients); a and b, regression equation coefficients y = a + bx; SEE (standard error of estimate); r2, coefficient of determination. n.s. not significant;*** p < 0.001. Male + Female(Total); Male(M) and Female(F).

These regression equations allow the construction of a basic table of predictions to be constructed according to arch and sex, as showed in Table 5.

**Table 5:** **Prediction table for the Spanish population based on regression equations**

**LI (mm)**	**UCPM(mm)**			**LCPM(mm)**		
	**T**	**M**	**F**	**T**	**M**	**F**
19	20.32	20.66	20.23	19.72	20.07	19.45
19.5	20.53	20.87	20.45	19.95	20.29	19.68
20	20.74	21.08	20.66	20.17	20.51	19.91
20.5	20.95	21.29	20.88	20.40	20.73	20.14
21	21.16	21.50	21.09	20.62	20.95	20.37
21.5	21.37	21.71	21.31	20.85	21.17	20.60
22	21.58	21.92	21.52	21.07	21.39	20.83
22.5	21.79	22.13	21.74	21.30	21.61	21.06
23	22.00	22.34	21.95	21.52	21.83	21.29
23.5	22.21	22.55	22.17	21.75	22.05	21.52
24	22.42	22.76	22.38	21.97	22.27	21.75
24.5	22.63	22.97	22.60	22.20	22.49	21.98
25	22.84	23.18	22.81	22.42	22.71	22.21
25.5	23.05	23.39	23.03	22.65	22.93	22.44
26	23.26	23.60	23.24	22.87	23.15	22.67
26.5	23.47	23.81	23.46	23.10	23.37	22.90

Lower Incisors (LI), Upper and Lower Canines and Premolars (UCPM, LCPM) tooth sizes. Male + Female(Total); Male(M) and Female(F).

Table 6 presents a comparison of regression constants among different populations including the own sample.

**Table 6:** **Regression parameters for predicting UCPM and LCPM tooth sizes in each arch and per sex**

**Study**	**Population**	**Sample (n)**	**Sex**	**Arch**	**Constants**				
					**r**	**a**	**b**	**SEE**	**r** ^**2**^
**Tanaka and Jhonston** [3]	**North American Whites**	**T = 506**	**M + F**	**Mx**	0.63	10.41	0.51	0.86	0.35
			**M + F**	**Mb**	0.65	9.18	0.54	0.85	0.42
**Al khadra** [9]	**Saudi Arabian**	**T = 34**	**M + F**	**Mx**	0.65	7.20	0.63	-	0.42
			**M + F**	**Mb**	-	8.60	0.55	-	0.40
**Diagne et al** [14]	**Senegalese**	**25(M) + 25(F)**	**M + F**	**Mx**	0.68	9.87	0.53	0.71	0.46
		**=50**	**M + F**	**Mb**	0.73	5.67	0.70	0.81	0.54
**Frankel and Benz** [21]	**Black Americans**	**39(M) + 41(F)**	**M + F**	**Mx**	0.65	10.18	0.52	0.87	0.42
		**T = 80**	**M + F**	**Mb**	0.70	8.30	0.64	0.94	0.49
**Jaroontham and Godfrey** [12]	**Northeastern Thai**	**215(M) + 215(F)**	**M + F**	**Mx**	0.60	11.87	0.47	0.84	0.36
		**T = 430**	**M + F**	**Mb**	0.64	10.30	0.50	0.82	0.41
**Melgaco et al** [15]	**White Brazilians**	**240(M) + 223(F)**	**F**	**Mx**	0.69	9.20	0.55	-	0.48
		**=500**	**M**	**Mb**	0.70	8.90	0.58	-	0.50
**Nourallah et al** [13]	**Syrians**	**320(M) + 280(F)**	**M + F**	**Mx**	0.67	9.87	0.50	0.79	0.45
		**T = 600**	**M + F**	**Mb**	0.68	9.32	0.55	0.83	0.46
**Uysal et al** [17]	**Turkish**	**100(M) + 128(F)**	**M + F**	**Mx**	0.99	4.07	0.76	0.01	0.98
		**T = 228**	**M + F**	**Mb**	0.99	3.74	0.75	0.01	0.98
**Paredes et al. (this study)**	**Spanish**	**169(M) + 190(F)**	**M + F**	**Mx**	0.574	12.34	0.42	0.87	0.330
		**T = 359**	**M + F**	**Mb**	0.587	11.17	0.45	0.91	0.345
**Bherwani and Fida** [19]	**Pakistani**	**100(M) + 100(F)**	**M + F**	**Mx**	0.65	8.56	0.54	0.79	0.42
		**T =200**	**M + F**	**Mb**	0.59	10.52	0.48	0.82	0.35
**Yuen et al** [10]	**Hong Kong Chinese**	**61(M) + 51(F)**	**M + F**	**Mx**	0.72	8.13	0.63	0.74	0.52
		**T = 112**	**M + F**	**Mb**	0.73	7.74	0.61	0.71	0.53
**Abu Alhaija et al** [23]	**Jordanian**	**130(M) + 96(F)**	**M + F**	**Mx**	0.57	10.55	0.53	0.99	0.32
		**T = 226**	**M + F**	**Mb**	0.59	9.41	0.56	0.99	0.35
**Philip NI et al** [18]	**Indian**	**300(M) + 300(F)**	**M + F**	**Mx**	0.65	7.29	0.65	0.43	0.76
		**T = 600**	**M + F**	**Mb**	0.67	5.85	0.69	0.45	0.75
**Chan et al** [11]	**Asian Americans**	**T = 201**	**M + F**	**Mx**	0.64	8.19	0.63	0.90	0.41
			**M + F**	**Mb**	0.66	7.46	0.62	0.85	0.44
**Tahere et al** [22]	**Iranian**	**25(M) + 25 F)**	**M + F**	**Mx**	0.53	11.06	0.45	0.80	0.28
		**T = 50**	**M + F**	**Mb**	0.70	6.42	0.64	0.70	0.49

r (Pearson linear regression coefficients); a and b, regression equation coefficients y = a + bx; SEE, r^2^, coefficient of determination. Male + Female (Total); Male(M) and Female(F) of different populations. Mx (maxilla) and Mb (mandible).

**Figure 1:**
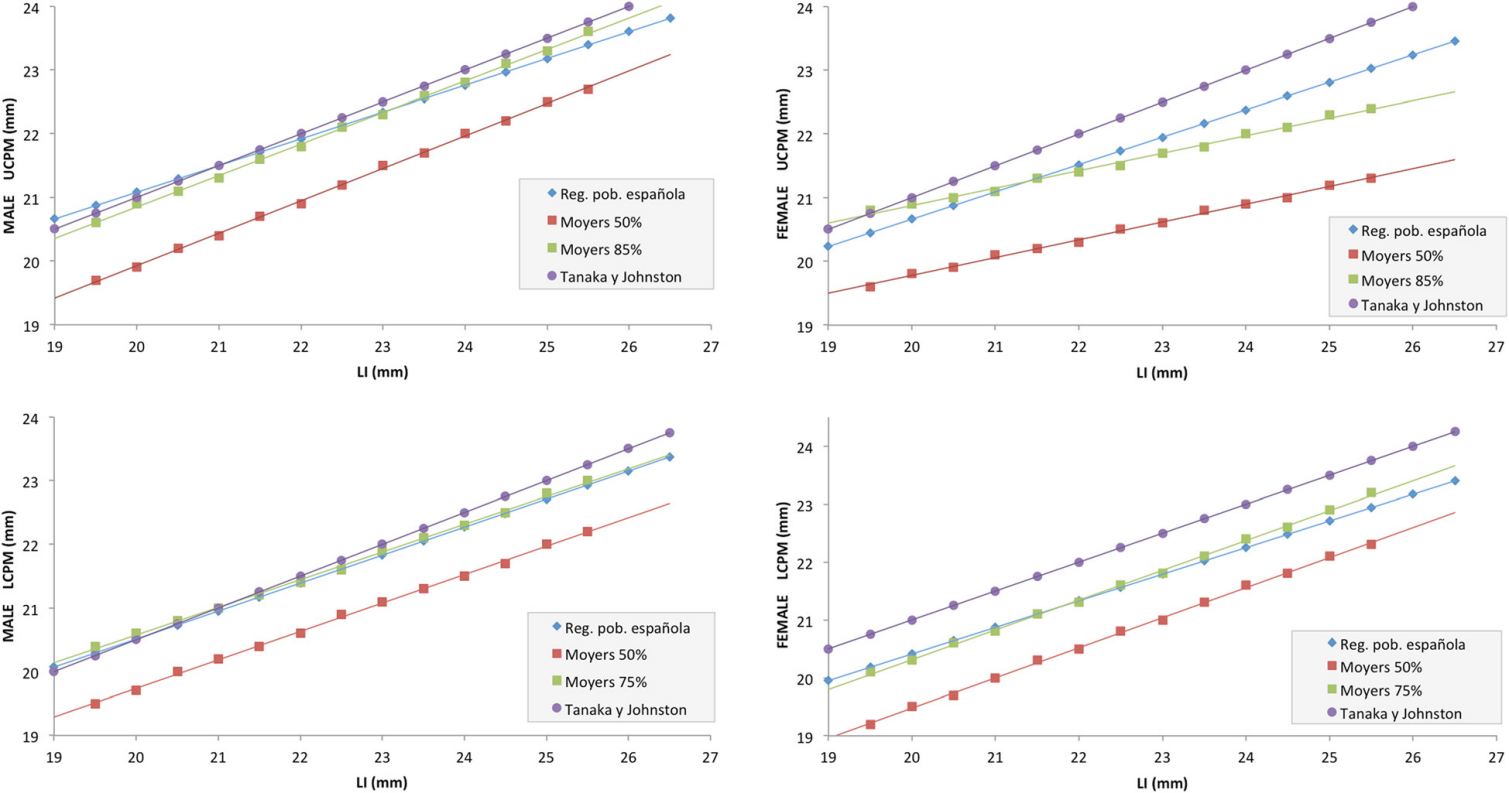
**UCPM y LCPM predictions for males and females respectively using the 50% Moyers method, the 85% Tanaka-Johnston method and the estimated regression line.**

Finally, in Figure 1, predictions of the three methods are compared; estimated regression lines, Moyers tables at 50% and at 85%, and the Tanaka-Johnston Rule.

## Discussion

No statistically significant differences between males and females were found for LI mesiodistal size, unlike those of UCPM and LCPM, where males presented statistically higher mesiodistal sizes than females. These results for the LI agree with studies published on Chinese [10] and Indian [18] populations, but are contrary to studies on Brazilian [15], Pakistani [19], Turkish [17] or Thai [12] populations, where statistically significant differences were found in LI sizes, as well as in UCPM and LCPM sizes.

Moyers Tables [2] are classified from the 95th to 5th prediction level. The most practical level from the clinical point of view is the 75th level, although in theory the 50th level of probability should be used, as any error will be evenly distributed in either direction.

Taking the prediction of upper arch at total level into account, as showed in Table 2, all differences are positive, indicating that the Moyer’s Tables tend to underestimate the real values of the UCPM in the sample of Spanish individuals, apart from for the 95% level. Practically, statistically significant differences were found at all confidence levels apart from those for males at the 85% confidence level, where no statistically significant difference was found and thus, homogeneity can be accepted. In females, homogeneity may be verified for a slightly higher percentile of around 90%.

Regarding the lower arch, the applicability of Moyer’s [2] tables is only useful for the 75th percentile, both at total level and for each sex where no statistically significant differences were found, contrary to all the other levels evaluated. Generally speaking, it can be stated that Moyer’s values systematically tend to underestimate real values in the Spanish population. These results coincide with studies in a Brazilian population [15] at the 50th and 75th percentile levels and in a Jordanian population [23] except for the 65th and 75th percentile for female subjects and the 85th for male subjects, in a Pakistani population [19] and for South Indian children [18]. On the other hand, Saudi Arabian population [9] studies found that the recommended 75th percentile overestimated the mesiodistal sizes of canine and premolars.

Estimates obtained from the Tanaka-Johnston equation [3] produced several equally disputable results as both at the mandible and maxilla level, in both males and females, the predictions tended to overestimate the real UCPM and LCPM sizes of individuals, with all the discrepancies found being statistically significant. These results coincide with those of other authors on Iranian [22] and Pakistani [19] populations, whereas they differ from those on a Jordanian population [23], where the regression equations underestimated the real teeth value.

Finally, as it can be observed, adjustment for regression presents quite similar accuracy regardless of the arch and sex of patients, even though it tends to be slightly greater among females, as showed in other studies.

Pearson’s linear correlation coefficient (r) ranges from between 0.57 and 0.61 depending on combinations. Therefore, the proportion of total variance of the UCPM or LCPM variable explained by the LI, ranges from between 33.3% and 37.9%. SSE denotes the standard error in the predictions that were obtained with the corresponding regression equation. This fluctuates in the range of 0.84-0.91. The above mentioned equations presented a degree of accuracy similar to studies performed on Saudi-Arabians [9], Hong Kong Chinese [10], Asian Americans [11], North-Eastern Thais [12], Syrians [13], Senegalese [14], white Brazilians [15], Indian [18], Pakistani [19], black Americans [21], Iranian [22] and Jordanian [23] individuals, but lower than for the Turkish population [17] as can be seen in Table 6. The reason may lie in a greater dispersion of the CPM spaces belonging to the dental morphology of the individuals of the studied group. Likewise, it can be observed that the mandibular arch obtained higher “r” values than the maxillar in almost all of the studied populations. However, the validity of some data can be questioned due to the sample sizes of some studies.

Analysing Figure 1, the part corresponding to the UCPM for males, it can be observed that the line for the regression equation is the one better fitting the reality of the sample. It can be seen that up to LI values of around 23.5-24 mm, the predicted UCPM is greater than that estimated by Moyer’s [2] at 85% and by Tanaka-Johnston [3]. However, from 24 mm upwards the trend is reversed. In contrast, predictions for females present a clearly different pattern from those of males. With the exception of the lowest LI values, the predictions obtained with the regression model are situated at an intermediate level between the underestimate of Moyers [2] at 85% and the overestimate of Tanaka and Johnston [3].

For predictions of LCPM in males, the different methods present similar estimation lines to those of the UCPM. For predictions of LCPM in females, the graphic is again very similar to that of the UCPM, with the exception of the Moyers predictions at 75% that graphically present a very homogenous line to that of the regression line drawn up for the Spanish population. It can also be observed in the four graphics that Tanaka-Johnston method tends to overestimate more than other methods, UCPM and LCPM values.

Teeth size differ among people of various biological origins, tooth sizes differ. Some of the most used methods to predict the size of unerupted posterior permanent teeth were developed for North Americans ancestry, so the applicability and the effectiveness of these methods in others populations are inadequate, hence the need to draw up tables for each population.

## Conclusions

The conclusions of the study are:

1. Predictions of UCPM and LCPM sizes from LI for the Spanish ancestry population are evaluated quite differently depending on the used method.
2. Moyer’s tables tend to underestimate UCPM and LCPM in Spanish ancestry subjects, only being of use at the 75% level percentile for the mandible, both in males and females, and for the maxilla at the 85% and 90% level percentile for males and females respectively.
3. Estimates obtained from the Tanaka-Johnston equation tend to overestimate UCPM and LCPM sizes in Spanish ancestry subjects.
4. The equations for estimating the combined width of the unerupted canine and premolars are:
  $$ \begin{array}{l}\mathrm{Males}:\mathrm{UCPM}=12.68+0.42\mathrm{L}\mathrm{I}\ \mathrm{and}\ \mathrm{LCPM}=11.71+0.44\mathrm{L}\mathrm{I}\\ {}\mathrm{Females}:\mathrm{UCPM}=12.06+0.43\mathrm{L}\mathrm{I}\ \mathrm{and}\ \mathrm{LCPM}=10.71+0.46\mathrm{L}\mathrm{I}\end{array} $$

## References

[1] Irwin RD, Herold JS, Richardson A (1995). Mixed dentition analysis: a review of methods and their accuracy. Int J Pediatr Dent.

[2] Moyers RE (1973). Handbook of Orthodontics.

[3] Tanaka MM, Johnston LE (1974). The prediction of the size of unerupted canines and premolars in a contemporary orthodontic population. J Am Dent Assoc.

[4] Nance HN (1947). The limitation of Orthodontic treatment I. Mixed dentition diagnosis and treatment. Am J Orthod Oral Surg.

[5] De Paula S, Almeida de Oliveira MA, Lee PCF (1995). Prediction of mesiodistal diameter of unerupted lower canines and premolars using 45° cephalometric radiography. Am J Orthod Dentofacial Orthop.

[6] Hixon EH, Oldfather RE (1958). Estimation of the sizes of unerupted cuspid and bicuspid teeth. Angle Orthod.

[7] Bishara SE, Staley RN (1984). Mixed dentition mandibular arch analysis. Am J Orthod.

[8] Staley RN, Kerber RE (1980). A review of the Hixon and Oldfather mixed dentition prediction Method. Am J Orthod Dentofacial Orthop.

[9] Al-Khadra BH (1993). Prediction of the size of unerupted canines and premolars in a Saudi Arab population. Am J Orthod Dentofacial Orthop.

[10] Yuen KK, Tang EL, So LL (1998). Mixed dentition analysis for Hong-Kong Chinese. Angle Orthod.

[11] Lee-Chan S, Jacobson BN, Chwa KH, Jacobson RS (1998). Mixed dentition analysis for Asian Americans. Am J Orthod Dentofacial Orthop.

[12] Jaroontham J, Godfrey K (2000). Mixed dentition space analysis in a Thai population. Eur J Orthod.

[13] Nourallah AW, Gesch D, Khordaji MN, Splieth C (2002). New regressions equations for predicting the size of unerupted canines and premolars in a contemporary population. Angle Orthod.

[14] Diagne F, Diop-Ba K, Ngom PI, Mbow K (2003). Mixed dentition analysis in Senegalese population: elaboration of prediction tables. Am J Orthod Dentofacial Orthop.

[15] Melgaço CA, Araújo MT, Ruellas AC (2006). Applicability of three tooth size prediction methods for white Brazilians. Angle Orthod.

[16] Paredes V, Gandia JL, Cibrian R (2006). A new, accurate and fast digital method to predict unerupted tooth size. Angle Orthod.

[17] Uysal T, Bascifici FA, Goyenc Y (2009). New regression equations for mixed-dentition arch analysis in a Turkish sample with no Bolton tooth-size discrepancy. Am J Orthod Dentofacial Orthop.

[18] Philip NI, Prabhakar M, Arora D, Chopra S (2010). Applicability of the Moyers mixed dentition probability tables and new prediction aids for a contemporary population in India. Am J Orthod Dentofacial Orthop.

[19] Bherwani AK, Fida M (2011). Development of a prediction equation for the mixed dentition in a Pakistani sample. Am J Orthod Dentofacial Orthop.

[20] Paredes V, Williams FD, Cibrian R, Williams FE, Meneses A, Gandia JL (2011). Mesiodistal sizes and intermaxillary tooth-size ratios of two populations; Spanish and Peruvian. A comparative study. Med Oral Patol Oral Cir Bucal.

[21] Frankel HH, Benz EM (1986). Mixed dentition analysis for black Americans. Pediatr Dent.

[22] Nik Tahere H, Majid S, Fateme M, Kharazi Fard K, Javad M (2007). Predicting the size of unerupted canines and premolars of the maxillary quadrants in an Iranian population. J Clin Pediatr Dent.

[23] Abu Alhaija ES, Qudeimat MA (2006). Mixed dentition space analysis in a Jordanian population: comparison of two methods. Int J Paediatr Dent.

[24] Paredes V, Gandia JL, Cibrian R (2005). New, fast, and accurate procedure to calibrate a 2-dimensional digital measurement method. Am J Orthod Dentofacial Orthop.

